# Role of Plant Phytochemicals: Resveratrol, Curcumin, Luteolin and Quercetin in Demyelination, Neurodegeneration, and Epilepsy

**DOI:** 10.3390/antiox13111364

**Published:** 2024-11-07

**Authors:** Mikołaj Grabarczyk, Weronika Justyńska, Joanna Czpakowska, Ewa Smolińska, Aleksandra Bielenin, Andrzej Glabinski, Piotr Szpakowski

**Affiliations:** 1 Medical Faculty, Medical University of Lodz, 90-419 Lodz, Poland; mikolaj.grabarczyk@stud.umed.lodz.pl (M.G.); weronika.justynska@stud.umed.lodz.pl (W.J.); ewa.smolinska@stud.umed.lodz.pl (E.S.); aleksandra.bielenin@stud.umed.lodz.pl (A.B.); 2 Department of Neurology and Stroke, Medical University of Lodz, Zeromskiego 113 Street, 90-549 Lodz, Poland; joanna.czpakowska@umed.lodz.pl (J.C.);

**Keywords:** polyphenols, resveratrol, curcumin, quercetin, luteolin, demyelinating diseases, neurodegenerative diseases, epilepsy

## Abstract

Polyphenols are an important group of biologically active compounds present in almost all food sources of plant origin and are primarily known for their anti-inflammatory and antioxidative capabilities. Numerous studies have indicated their broad spectrum of pharmacological properties and correlations between their increased supply in the human diet and lower prevalence of various disorders. The positive effects of polyphenols application are mostly discussed in terms of cardiovascular system well-being. However, in recent years, they have also increasingly mentioned as prophylactic and therapeutic factors in the context of neurological diseases, being able to suppress the progression of such disorders and soothe accompanying symptoms. Among over 8000 various compounds, that have been identified, the most widely examined comprise resveratrol, curcumin, luteolin and quercetin. This review focuses on in vitro assessments, animal models and clinical trials, reflecting the most actual state of knowledge, of mentioned polyphenols’ medicinal capabilities in epilepsy, demyelinating and neurodegenerative diseases of the central nervous system.

## 1. Introduction

Among the heterogenous group of multiple phytochemicals, plant polyphenols are considered the most abundant group. Their chemical structure contains at least two phenyl rings with one or more hydroxyl substituents. There are many possible ways to classify those vegetal secondary metabolites [1]. The most common classification systems focus on the structure of the carbonic skeleton and the number of mentioned basal elements and assign specific compounds to one of the four main subclasses: flavonoids, phenolic acids, lignans and stilbenes. Flavonoids divide further into flavonols, flavanols, flavanones, flavones, isoflavones and anthocyanidins. Similarly, phenolic acids are a combination of two smaller groups derived from hydroxybenzoic acid and hydroxycinnamic acid [2]. Not all polyphenols fulfill the criteria of the mentioned subgroups and are then referred to as “other polyphenols”.

Plants produce and accumulate polyphenols in stress conditions to mitigate harmful effects caused by environmental factors like infections or ultraviolet (UV) radiation. These compounds take part in the modulation of the life cycle of the plant cell, photosynthesis, hormonal regulation or protection against various pathogens [3,4]. Today, more than 8000 different polyphenols have been described [5]. This review focuses on four representatives of those phytochemicals: resveratrol, curcumin, luteolin and quercetin. Chosen compounds constitute an important fraction of dietary polyphenol intake, especially among people on a Mediterranean diet (MD). MD is considered one of the most effective nutrition patterns in the prevention and alleviation of numerous disorders, including cardiovascular diseases, cancer and autoimmunity. Furthermore, curcumin is a common spice, broadly used in different types of natural Asian diets, which is also associated with a lower prevalence of lifestyle diseases. As these polyphenols are of great interest to researchers, the amount of available tests and examination results allows for the creation of complex but at the same time accurate profiles of their action and potential in medical applications [6,7,8,9]. Resveratrol belongs to the stilbenes group and was first discovered in the roots of *Veratrum grandiflorum*. Grapes, peanuts, blueberries, cranberries and their byproducts are the food sources with the highest resveratrol content. Although it is quite effectively absorbed from aliment, its half-life in plasma oscillates around 10–15 min. Humans tolerate doses not exceeding 4 g per day and the most common metabolites of this phytochemical include resveratrol-3-*O*-sulfate, resveratrol-4′-*O*-glucuronide and resveratrol-3-*O*-glucuronide [10]

Curcumin, a polyphenol responsible for the yellow pigment of the Indian spice turmeric (*Curcuma longa*), is also its main source. Chemically, curcumin does not belong to any of the four main polyphenols’ subclasses. However, it is an important element of the curcuminoids family in the “other polyphenols” group [11,12]. Curcumin possesses methoxy, hydroxyl, α, β-unsaturated carbonyl and diketone groups, which are most likely responsible for its biological activity [13].

Luteolin is one of the most bioactive flavonoids. Chemically it is described as tetrahydroxy flavone, which means that it exhibits two hydroxyl groups on each of the two benzene rings. Naturally, it is mostly encountered in glucoside form. Luteolin-3′-*O*-β-d-glucuronide and luteolin-3′-*O*-sulfate are its most abundant metabolites, present in human plasma [14]. Significant amounts of luteolin are present in tansy leaf, rooibos and chicory [15].

Quercetin can be isolated from white mulberry, tea plant or Welsh onion. Same as luteolin, it belongs to the flavonoids subclass [16]. Its antioxidant properties are attributed to the presence of hydroxyl and catechol groups in the benzene rings. The most common derivatives of quercetin take the form of glycosides and ethers [17].

Even though polyphenols are naturally produced by plants, their positive capabilities are not limited to this group of organisms. Numerous studies have proved that their application to animals and humans exerts anti-inflammatory and antioxidant influence on cells of almost all tissues and organs [18]. Those properties are exceptionally well described in relation to cardiovascular disease prevention. Adequate intake of polyphenols is associated with lower blood pressure, a more favorable serum lipid profile and decreased risk of type 2 diabetes [19]. They are also efficient in tumor prevention and treatment, as a lot of epidemiological studies indicate that there is a connection between polyphenols consumption and the risk of various cancer types. In turn, research performed on animal models and tissue cultures provides data proving that they are able to induce apoptosis of neoplastic cells and restrain molecular pathways associated with tumor growth and spread [20]. The beneficial role of polyphenols is also becoming more and more established in the prevention or potential treatment of neurological disorders. This is especially noticeable in terms of neurodegenerative diseases and tumors of the central nervous system (CNS). Supplementation with certain polyphenolic compounds may improve motor impairment in Alzheimer’s or Parkinson’s disease and mitigate cognitive deficits [21]. Less is known about polyphenols’ therapeutic capabilities for demyelinating disorders or epilepsy. However, the data currently available allow us to consider them as potential treatment-supporting factors, because of their suppressive effect on inflammation accompanying demyelination and their sedative effect on the severity and frequency of epileptic seizures [22,23]. The limiting factor, with respect to the use of polyphenols as drugs or dietary supplements, is their poor bioavailability. Despite their effective absorption at the level of the intestinal barrier, hepatic metabolism reduces the amount of examined phytochemicals entering the bloodstream to approximately one percent of the initial dose. Nevertheless, a certain percentage of emerging metabolites retain pharmacological activity and exert biological responses [24]. When these compounds are used in the treatment of CNS diseases, there is also the issue of penetration through the blood–brain barrier (BBB). However, this barrier is also at least partially permeable for this kind of phytochemicals, as it is possible to detect dietary polyphenols in cerebrospinal fluid (CSF) [25]. The bioavailability of polyphenols may be raised using food or pharmaceutical engineering. Nanocomplexation with food biopolymers, gelation, complex coacervation and pH-shifting are some of the techniques used in order to prevent enzymatic degradation of polyphenols and promote absorption through biological barriers [26,27].

The main objective of this review was to summarize the most important reports concerning the potential role of selected polyphenols in the prevention and treatment of neurological disorders.

## 2. Polyphenols’ Role in Treatment of Demyelinating Diseases

Demyelination is a pathological process of myelin loss, that hinders the function of numerous nerve centers and peripheral, nervous pathways and underlies a complex group of neurological disorders collectively known as demyelinating diseases. Such disorders may be classified depending on the localization of affected regions in the nervous system, advancement of symptoms and molecular or histopathological basis of the disease [28,29]. Demyelination can be caused by many different factors. Some sorts of demyelinating diseases are hereditary and genetically determined by functional abnormalities or pathological storage of metabolites in oligodendrocytes or other tissues accompanying neurons. Today they are classified as a leukodystrophy group [30]. Others are paraneoplastic syndromes, accompanying specific neoplasms, or manifestations of viral infections, like progressive multifocal leukoencephalopathy (PML) caused by the John Cunningham (JC) virus. Demyelination may be also a result of iatrogenic damage connected with rare side effects of some medications, mostly biological ones affecting immunological transmitters, or aggressive treatment of hyponatremia resulting in central pontine myelinolysis [31,32,33]. However, the most abundant and most common group of demyelinating disorders are idiopathic inflammatory demyelinating diseases that include neuromyelitis optica spectrum disorders (NMOSD), myelin oligodendrocyte glycoprotein antibody disease (MOGAD), acute disseminated encephalomyelitis (ADEM) and multiple sclerosis (MS) with its variants as their dominant representative. The exact cause of those illnesses is unknown or very complex, with numerous genetical and environmental factors contributing to the breakage of immunological tolerance of the organism’s own cerebral cells and components [34]. Actual treatment strategies for such disorders focus on the modulation and inhibition of abnormal and excessive inflammatory responses through varied pharmaceuticals affecting elements of humoral and cellular immunity [35]. Interventions with quick, strong, non-selective and wide-ranging immunosuppressive effects, like corticosteroid injections, intravenous immunoglobulins or plasma exchanges, are mostly used in relapses of the disease [36]. For MS there is a wide spectrum of medicines used in long-term maintenance of the illness with complex mechanisms of action. Despite such a diverse set of available pharmaceuticals, the search for new treatment forms still continues. As the inflammation in those types of demyelinating disorders is complex and engages varied types of effector cells, ranging from plasma cells and T CD4^+^ cells to astrocytes and microglia, the most promising potential medications shall possess pleiotropic, multi-faceted anti-inflammatory capabilities to effectively counteract immunological response to organism’s own myelin elements and other cell fractions associated with them [34]. Such capabilities are displayed by polyphenolic compounds, whose ability to soothe inflammation and inflammation-based diseases have been well reported in numerous studies based on in vitro and in vivo models as well as clinical trials, including ones that concern demyelination [37,38,39]. Plant polyphenols may affect the secretory response of antigen-stimulated cells, lowering cytokines and chemokines release, accompanied by silencing of intracellular pathways associated with lymphocyte maturation and differentiation. Antioxidative capacities allow for protection against oxidative stress accompanying inflammatory response [40]. They also have an influence on the permeability of biological membranes and barriers, which in terms of demyelinating disorders is especially important regarding BBB [41]. Their therapeutic potential is not limited to effects exerted on immunological interactions, as some studies indicate that polyphenolic compounds may promote the maturation of oligodendrocytes and support the formation of myelin. Studies on animal models of multiple sclerosis indicate that polyphenols in monotherapy or as add-on drugs decrease motor impairment caused by demyelination and lower the severity of illness [42,43]. Their main flaw is poor bioavailability, and actual methods for efficient polyphenol delivery are underdeveloped. The most valuable solutions are chemical structure modifications of bioactive compounds or the use of nanocarriers [44,45]. Despite such wide-ranging therapeutic influence, all quoted capabilities are too weak to consider known polyphenolic compounds as stand-alone drugs for demyelinating disorders. However, considering their lack of toxic effects, they could make an efficient addition to currently used treatment regimens in the form of combined therapy.

### 2.1. Resveratrol’s Impact on Demyelinating Diseases

**Figure 1:**
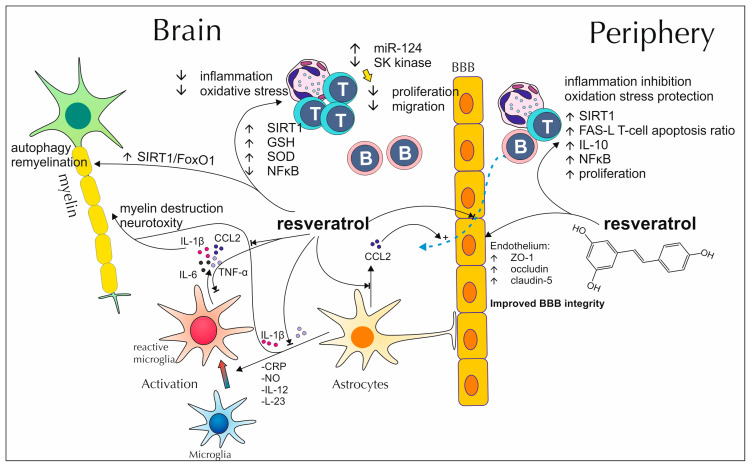
Resveratrol’s protective role in demyelination. Resveratrol has been found to modulate the secretory activity of astrocytes and microglial activation by downregulating the release of IL-1β, IL-6, TNF-α, CRP, NO, IL-12, and IL-23, which in turn provides myelin protection and decreases inflammation. Activation of the SIRT1 pathway by resveratrol supports remyelination, protects against oxidative stress and suppresses inflammation. Resveratrol increases the expression of tight junction proteins (such as zonula occludens, occludin and claudin-5) in brain endothelial cells, enhancing blood–brain barrier (BBB) integrity and reducing leukocyte recruitment. In the periphery, resveratrol inhibits inflammatory reactions by increasing IL-10 production in leukocytes and inducing FAS-L-dependent apoptosis.

Resveratrol’s direct impact on mechanisms associated with myelin’s development and resistance to myelin damage was not widely investigated in in vitro research. Most data refer to its general immunomodulatory potential, with special consideration of inflammatory mediators and effector cells of the greatest importance in demyelinating disorders pathology (Figure 1). Excessive cytokine production underlies chronic inflammation within structures of the CNS and is one of the core factors responsible for multiple sclerosis development and progression. Resveratrol alters the cytokine production profiles of various glial cells. Reactive, human microglia, stimulated with this polyphenol, show reduced secretion of interleukin (IL)-1 and IL-6, two of the most versatile pro-inflammatory cytokines [46]. Similarly, murine microglia exposed to resveratrol decreased IL-1 and IL-6 release as also tumor necrosis factor α (TNF-α) and chemokine ligand (CCL)-2, which confirms the lower pro-inflammatory potential of these cells [47]. Moreover, Wight et al. noted that in lipopolysaccharide (LPS)-exposed astrocytes derived from mice, resveratrol mitigates astrocytes’ pro-inflammatory response by decreasing C-reactive protein (CRP), nitric oxide (NO), IL-1β, TNF-α, CCL2, IL-12p40 and IL-23 production. This, in turn, may not only protect from enormous activation of glia and the development of neurotoxic astrocytes but also counteracts the differentiation of Th1 and Th17 cells, whose increased activity is related to worse and more severe clinical manifestations of experimental autoimmune encephalomyelitis (EAE), the animal model of MS [48]. Hostenbach et al. noted in their study that resveratrol may as well decrease the expression of endothelin-1 (ET-1) in human astrocytoma cell lines. The mentioned peptide, highly produced by reactive astrocytes of demyelinated plaques found in the brains of patients suffering from MS, is postulated to contribute to this disease pathology by modifying BBB permeability and causing cerebral hypoperfusion [49]. Resveratrol intracellularly activates sirtuin 1 (SIRT-1) a signaling protein that normally decreases pro-inflammatory cytokines production on transcription level and counteracts the effect of microRNA (miR)-132 in B cells [50]. In a study conducted on a murine model of cuprizone-induced MS, an addition of resveratrol to the animals’ diet resulted in the repression of an overly intensified, inflammatory nuclear factor κB (NF-κB) signaling pathway in brain tissue. Improvement was also visible in the state of mechanisms involved in protection from oxidative stress, as polyphenol-fed mice presented higher glutathione (GSH) levels and greater superoxide dismutase (SOD) activity. This was reflected in the alleviated loss of myelin, stated in histological analyses, and better results achieved in balance and locomotor coordination tests [51]. Samy et al. suggest that this positive influence on remyelination processes may come from the stimulation of autophagy mechanisms, achieved through SIRT1/forkhead box protein O1 (FoxO1) pathway activation, as defective removal of myelin prevents the remyelination process from proceeding properly [52]. Similar results were acquired by Gandy et al. in an EAE model, where resveratrol proved capable of soothing the motor impairment suffered by mice, decreased mononuclear cell infiltration into the brain and lowered blood levels of TNF-α and interferon-γ (IFN-γ). This was accompanied by changes in encephalitogenic CD4^+^ T cells’ microRNA profile, main upregulation of miR-124, resulting in lower expression of serum/glucocorticoid regulated kinase 1 (SK1) and silencing of SK1-dependent signaling for cell proliferation and migration. Consequently, an increased apoptosis ratio was also observed. It is worth noting, that this activity was specific for brain-derived cells and not detectable among peripheral immune cells [53]. Singh et al. imply that resveratrol-induced apoptosis among activated T cells might be initiated by the activation of the aryl hydrocarbon receptor (AhR) and estrogen receptor (ER), which in turn, results in the upregulation of death receptor–Fas and its ligand–FasL presence on the cell membrane. A reduced expression of anti-apoptotic genes (B-cell lymphoma-extra-large (Bcl-xl) and second mitochondria-derived activator of caspases (Smac)) was also observed, suggesting the involvement of the mitochondrial apoptosis pathway [54]. Other experiments, conducted on EAE mice, show that resveratrol may attenuate the development of this disease by counteracting the loss of tight junction proteins, most notably occludin, claudin-5 or zonula occludens-1 (ZO-1). Simultaneously, it has a negative impact on the expression of adhesion molecules involved in diapedesis intercellular adhesion molecule-1 (ICAM-1) and vascular cell adhesion protein-1 (VCAM-1). Thanks to these capabilities, it contributes to the improvement of BBB integrity and impairs the peripheral blood immune cells inflow into the structures of CNS. Schindler et al., report resveratrol’s potential to mitigate axonal loss and neuronal damage in optic nerves and spinal cord during EAE although, suppression of neuroinflammation or demyelination was not observed [55,56]. Similar results were acquired in a study using resveratrol in the form of nanoparticles. In spite of the resveratrol administration, the severity of inflammation within the spinal cord and optic nerves was unaffected; however, administration of resveratrol in nanoparticles resulted in reduced retinal ganglion cell loss and prevented more severe scores of EAE severity [57]. Imler Jr. et al. postulate that the explanation behind such results comes from resveratrol-altered secretory profiles of immune cells penetrating CNS. This polyphenol promotes IL-17^+^IL-10^+^ T cells and CD4^−^ IFN-γ^+^ cells development, these in turn, protect against more pathogenic forms of T cells, that contribute to MS pathology [58]. A different study using a chronic mouse model of MS (animals immunized with myelin oligodendrocyte glycoprotein (MOG) peptide 35–55) showed that resveratrol, when administered as premedication for at least 30 days before immunization, postpones EAE’s onset for a few days [59]. Likewise, it may enhance the effects of other therapeutic interventions in this model, as the addition of resveratrol to mouse bone marrow mesenchymal stem cells (mBM-MSC) treatment allows better clinical scores and higher reduction in inflammation advancement within the spinal cord than in uncombined treatment [60]. On the other hand, Sato et al. observed in their experiments that enriching mice’s diet with this stilbenoid, in a specific time window after the EAE or Theiler’s murine encephalomyelitis virus-induced demyelinating disease (TMEV-IDD) have been already induced, results in exacerbation of neuroinflammation’s severity within the spinal cord [61]. Such observations suggest a generally beneficial impact of resveratrol in demyelinating diseases; however, its potential to exacerbate disease symptoms when administrated after disease onset should be more intensively investigated and considered for restricted consumption in humans after disease onset. In a study conducted by Riccio et al. Resveratrol was used as one of the dietary supplements, next to vitamin D, omega-3 acids and numerous oligo-elements, implemented into the diet of patients suffering from MS. As there were many nutritional factors affecting patients state, it is hard to estimate this polyphenol’s exact role in acquired results. Although, applied dietary modification led to a decline in recognized inflammatory state markers and serum gelatinase levels, most notably matrix metalloproteinase-9 (MMP-9). Apart from this, no changes in neurological state or basic biochemical analyses, like lipid profile and hepatic or renal parameters, were found among patients [62].

### 2.2. Curcumin’s Impact on Demyelinating Diseases

Common pathogenic processes in MS are chronic inflammation and demyelination within the CNS. The main factors responsible for sustaining those processes are inflammatory cytokines secreted by both, immune system cells and nervous system cells, especially astrocytes and microglia. Curcumin, added to the growth medium, presents no toxicity toward cultured astrocytes [63]. It also possesses an ability to alter their secretory profile, which is particularly visible among reactive astrocytes, resulting in lower production of pro-inflammatory cytokines TNF-α, IL-2, IL-6, IFN-γ and IL-17A and decreased release of MMP-9 an enzyme postulated as an MS activity biomarker [64]. Similar data, regarding the modulation of cell’s secretory capabilities, comes from studies performed on microglia cell cultures. Curcumin proves to inhibit inducible nitric oxide synthase (iNOS), IL-6 and TNF-α expression in reactive microglia. Moreover, it maintains the expression of anti-inflammatory IL-10 at a more native-like level. The observed impact is accompanied by suppression of the Axl/Jak2/Stat3 pathway, a molecular trail involved in polarization of the microglia into their proinflammatory and neurotoxic state [65]. Reports concerning congruous effects come also from studies regarding elements of the immune system, located outside the CNS. Peripheral blood leukocytes, isolated from spleens of EAE-suffering mice, present lower IL-12, IL-17 and IL-23, and increased IL-10 secretion after exposition to curcumin [66]. Another study, performed on CD4^+^ cells (Th0, Th1, Th17 and Treg) from EAE mice, shows that curcumin suppresses their activation, promotes apoptosis and decreases colony-formatting potential [67]. This polyphenol’s positive impact, concerning possible treatment of MS, is also expressed through its protective and pro-developmental capabilities towards myelin or myelin-related elements. Tegenge et al., using cocultures of neurons and microglia, demonstrated that curcumin may counteract NO-mediated axon degeneration [68]. Furthermore, it boosts oligodendrocyte progenitor cells maturation into more differentiated forms and upregulates secretion of myelin basic protein (MBP), a fundamental component of myelin structure. It also protects those cells from adverse effects exerted on their viability and maturation process by TNF-α [69]. Curcumin addition to the diet of EAE mice allows for partial recovery from illness, reflected in lower disease severity scores. Furthermore, it is capable of soothing EAE-induced body mass loss and cognitive impairment. Additionally, the inflammatory cell infiltration, especially CD3^+^ and CD4^+^, and demyelination within the spinal cord are reduced. In the cuprizone-induced MS model, the addition of curcumin contributes to lower microglia and astrocytes activation, soothes depletion of myelin content and provides protection from oxidative stress, as polyphenol-treated mice present lower malondialdehyde (MDA) levels and upregulated activity of antioxidant enzymes and compounds: SOD, catalase (CAT) and GSH in cuprizone induced demyelination [64,70,71]. Corresponding data were obtained in a study by Barzegarzadeh et al., from a rat, ethidium bromide–induced model of demyelination [72]. Motavaf et al. indicate that curcumin dendrosomal nanoparticles treatment, apart from counteracting demyelination, also promotes oligodendrogenesis from neuronal steam cells and oligodendrocytes progenitor cells, leading to more efficient regeneration of myelin [73]. Modified curcumin molecules seem to possess similar, or even stronger capabilities. In a study utilizing curcumin monoglucuronide, there was a general trend towards lower EAE severity scores, inflammation intensity and demyelination in the spinal cord among curcumin monoglucuronide (CMG)-fed mice, although it was not statistically significant. Additionally, connections between microbiota, curcumin supply and illness severity were found. CMG substantially decreased *Blautia gnavus* participation in mice faces, *Alistipes finegoldi* in ileal contents and *Burkholderia* spp. in ileal mucosa. All mentioned bacteria species correlated with worse EAE courses and higher severity scores [74]. Khosropour et al., Amiri et al. and Esmaeilzadeh et al. found that curcumin modulates cytokine gene expression within spinal cords, callosal commissures and spleens of EAE mice, increasing anti-inflammatory IL-4 and IL-10, transforming growth factor-β (TGF-β) and lowering pro-inflammatory IL-1, IL-6, TNF-α, IFN-γ, and IL-17. It also affects the expression of transcription factors specific for different subtypes of T cells, promoting GATA3 and FoxP3 and thus, Th2 and Treg lines development. Those fractions are considered protective in terms of demyelinating disorders genesis [75,76,77]. The opposite effect is exerted on the differentiation of Th17 cells, as curcumin downregulates the expression of cytokines and transcription factors responsible for inducing their development, notably TGF-β, IL-6, IL-21 and RAR-related orphan receptor gamma (RORγt). This subtype of T cells is recognized as one of the most important and crucial in the progression of MS and other autoimmune diseases [78]. In a study by Chearwae et al., T cells of EAE-suffering mice, treated with curcumin, demonstrated lower toll-like receptor (TLR)-4 and TLR-9 expression. Activation of those receptors contributes to exacerbation of T cell response and clinical manifestation of EAE [79]. Inhibiting influence, towards inflammatory cells presence within spinal cords, was reported regarding microglia, as their cell marker–transmembrane protein 119 (TMEM119) detectability falls in EAE mice treated with curcumin [65]. Polymerized nano curcumin (PNC) shows convergent regulatory capabilities towards pro- and anti-inflammatory factor secretion in the mentioned demyelinating disease model. Moreover, it may postpone EAE onset and peak by 3–5 days. It also protects against oxidative stress, which is visible in up-regulated transcription of mRNA for nuclear factor erythroid 2-related factor 2 (Nrf2), a signaling factor important in cellular, anti-oxidative pathways [80]. The development of EAE is also associated with unfavorable changes in the expression of other proteins, most notably NOGO-A, which takes part in the repression of axonal regeneration and microtubule-associated proteins 1A/1B light chain 3B II (LC3-II) with Beclin-1, involved in autophagic mechanisms. Among mice treated with curcumin, transcription patterns of their respective genes resemble the ones noted in unaffected mice [81,82]. Supplying curcumin, packed in particles imitating high-density lipoproteins, specifically increases its availability for monocytes and thus, prevents their penetration into the rodents’ brains. As they are responsible for inducing Th1 and Th17 cell influx, penetration of those cell subtypes is reduced as well [83].

**Figure 2:**
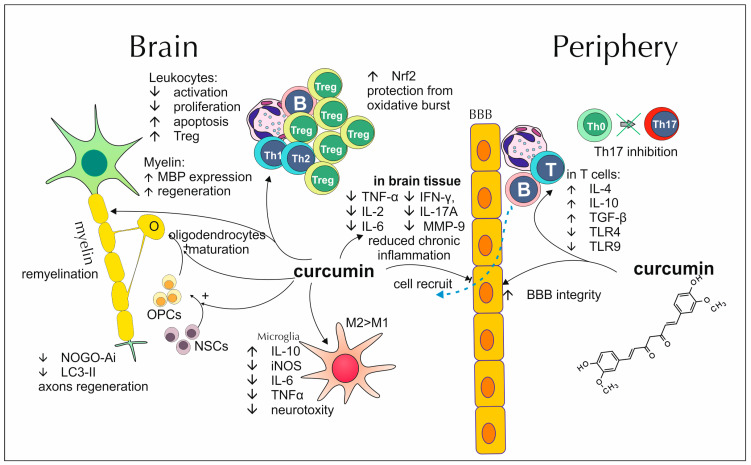
Curcumin’s protective role in demyelination. Curcumin acts in multiple ways: it modulates the activation state and secretory activity of microglia, promotes remyelination by supporting the maturation of oligodendrocytes from oligodendrocyte progenitor cells (OPCs) and neuronal stem cells (NSCs), and exhibits anti-inflammatory action by downregulating the release of pro-inflammatory cytokines from glial and immune cells (such as IL-17A and pro-inflammatory cytokines TNF-α, IL-2 and IL-6). It also promotes Treg differentiation, controls leukocyte proliferation and regulates Th17 differentiation. Furthermore, curcumin decreases neuroinflammation by enhancing blood–brain barrier integrity and modulating glial activation (by decreasing MMP-9 activity) and immune cell recruitment from the periphery.

In a randomized, controlled trial carried out by Petracca et al., curcumin’s therapeutic potential, as an add-on drug, was checked on patients with relapsing MS receiving IFN β–1a treatment. No significant differences between studied groups, in terms of new or enlarging lesions development and time to first relapse, were found. However, this lack of statistically significant distinctions may be due to a high dropout rate over the study course [39]. Great insight into curcumin’s potential as an MS-dedicated drug was acquired in a series of randomized, double-blind, placebo-controlled clinical trials performed by Dolati et al. The tested group consisted of patients in a relapsing-remitting multiple sclerosis state, with at least a 3-month-long history of IFN β–1a treatment. Curcumin, administered to the patients for 25 days, was encapsulated in nano-micelles to increase its bioavailability. Peripheral blood mononuclear cells, acquired from participants with MS, presented a disturbed profile of numerous peripheral blood cells’ micro-RNA. Specific roles of all those compounds are not fully discovered yet, although polyphenol treatment was associated with their expression patterns similar to the control group, thus restoring balance. Especially, the effect displayed towards miR-145 and miR-132 is worth noting, as their expression was the lowest in the curcumin-treated group when compared with both pre-treatment results and placebo-treated patients. This corresponded with weakened suppression of their target genes and sex-determining region Y-box 2 (Sox-2) and SIRT-1. Both micro-RNA compounds are considered mediators of the pro-inflammatory state and miR-145 serves as a specific marker of MS. Additionally, the IL-1β, IL-6, and IFN–γ amounts in serum were lower after curcumin application, also in comparison to the results from the placebo group. Nano-curcumin treatment, unlike placebo, was capable of decreasing the overall Th17 cell count in the peripheral blood of MS patients, as well as reducing PBMC’s expression of RORγt and IL-17 genes, reflected in lower secretion of same-name proteins. An inverse phenomenon occurred for Treg cells, as polyphenol admission increased their share in peripheral blood, as well as upregulated factor Foxp3, crucial for their development. This corresponded with raised production of IL-10 and TGF-β, visible in respective mRNA and protein levels. In all those studies, the patient’s EDSS score was decreased by curcumin treatment when compared to the initial examination and placebo-treated group [84,85,86,87]. The effects of curcumin on demyelinating processes are summarized in Figure 2.

### 2.3. Luteolin’s Impact on Demyelinating Diseases

**Figure 3:**
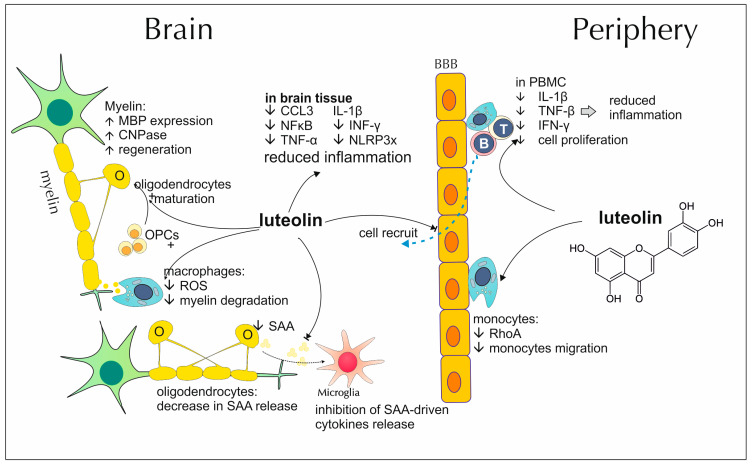
Luteolin’s protective role in demyelination. Luteolin decreases SAA release from oligodendrocytes and inhibits microglial activation. It regulates macrophage activation and ROS production, thereby protecting against myelin degradation. Moreover, luteolin supports myelin regeneration by activating oligodendrocyte precursor cells (OPCs) and promoting oligodendrocyte maturation. The anti-inflammatory action of luteolin is associated with decreased expression of cytokines and chemokines, as well as inhibition of NFκB signaling in brain tissue and mononuclear leukocytes in the periphery, limiting their recruitment to the brain and the autoimmune response.

Peripheral blood mononuclear cells (PBMCs), isolated from the blood of patients diagnosed with MS, demonstrate upregulated secretion of many proinflammatory cytokines like IL-1β or TNF-β. They also produce MMP-9, crucial for the immune system’s cell penetration through BBB. In a study by Sternberg et al., luteolin was able to lower the release of pro-inflammatory cytokines and reduce cell proliferation rate [88]. A different in vitro research, using PBMCs specific for alpha B-crystallin, considered an autoantigen in MS, showed that luteolin might decrease antigen-triggered secretion of IFN-γ [89]. A complex of palmitoylethanolamide and luteolin may promote myelination within the CNS. Facci et al. demonstrated that it enhances the differentiation of oligodendrocytes, derived from cortices of rat pups, which is accompanied by upregulated expression of two proteins important for myelin development and integrity–MBP and 2′,3′-Cyclic-nucleotide 3′-phosphodiesterase (CNPase) [42]. In a similar study, Skaper et al. noted that the MBP protein network, produced by oligodendrocyte progenitor cells incubated with the mentioned complex of luteolin, displays a more elaborate pattern, resembling the one observed among mature cells of this type. Moreover, they exhibit a rise in mRNA for proteolipid protein (PLP), another important structural factor of myelin, and decreased expression of apolipoprotein E (apoE), which is postulated as a potential intermediate compound during antigen presentation in MS development [43]. Macrophages are one of the main cell fractions contributing to MS pathology. They accumulate in CNS and secrete various factors that promote demyelination and are also capable of phagocytosing myelin themselves. Hendriks et al. conducted a series of tests with cultured, mice macrophages and isolated myelin, where they observed that flavonoids, and among them luteolin, suppress phagocytosis of myelin proteins by macrophages, without affecting their viability. Moreover, this is accompanied by a decrease in reactive oxygen species (ROS) production [90]. Serum amyloid A (SAA) is an acute-phase protein, produced mostly by the liver, although detectable in axonal myelin sheaths of cortex in MS as well. One of the possible sources of this protein, within the brain, is oligodendrocytes. It stimulates the production of typical inflammatory cytokines, particularly IL-1β and TNF-α, in cultured rat microglia and astrocytes. This effect is most likely exerted through the TLR4 receptor. Barbierato et al. presented that expression of SAA by oligodendrocytes in vitro may be decreased by incubation with palmitoylethanolamide and luteolin complex [91]. Mast cells may contribute to MS pathology by numerous interactions with other immune cells and alteration of BBB integrity. Their activation results in increased release of inflammatory cytokines, notably TNF-α, and IL-8, that stimulate T cells and promote their migration into the brain. Luteolin is capable of counteracting MBP-mediated mast cell activation as well as preventing further stimulation of T cells [90,92]. Demyelinated lesions, that form in the brains of MS-suffering patients, are characterized by a decreased ratio of mature oligodendrocytes and, at the same time, an increased share of apoptotic ones. Luteolin proves capable of reversing those changes, as in the lysolecithin demyelination model, mice fed with a diet containing this polyphenol presented higher participation of active oligodendrocytes and a greater percentage of MBP-rich areas, suggesting active remyelination [93]. Administration of luteolin to rats, previously immunized with MOG to induce EAE, improves clinical course and EAE severity. At the cellular level of brain tissue, it is manifested in decreased secretion of macrophage inflammatory protein 1α (MIP-1α) chemokine and lower level of NF-kB a transcription factor for MIP-1α and other proinflammatory mediators. In addition, the secretion of caspase-3, a protein capable of causing axon degenerative changes, was also decreased [94]. Moreover, a study conducted by Hendriks et al. reports inhibition of monocyte infiltration into the CNS. A postulated explanation for this specific effect may be luteolin’s capability to affect cytoskeletal regulatory components, as monocytes treated with luteolin presented fewer membrane protrusions. One such affected factor is Ras homolog family member (RhoA), a Guanosine-5′-triphosphatase (GTPase) crucial for the migration and diapedesis of monocytes [95]. Similar protective capabilities apply to palmitoylethanolamide and luteolin composite. EAE mice dosed with this compound develop the disease in a less serious form, as their clinical severity scores are lower when compared to placebo-treated groups. Evaluations of mRNA transcripts from cerebella and brainstems of such mice demonstrate a suppression in TNF-α, IL1-β, INF-γ and nod-like receptor family pyrin domain-containing 3 (NLRP3) inflammasome expression. Modulation applies as well to the expression profile of mRNA for various receptor proteins, most notably CD137, which is critical for signaling in both the induction and effector phase of EAE [96]. Figure 3 summarizes the most important modes of luteolin activity contributing to its anti-demyelinating properties.

### 2.4. Quercetin’s Impact on Demyelinating Diseases

**Figure 4:**
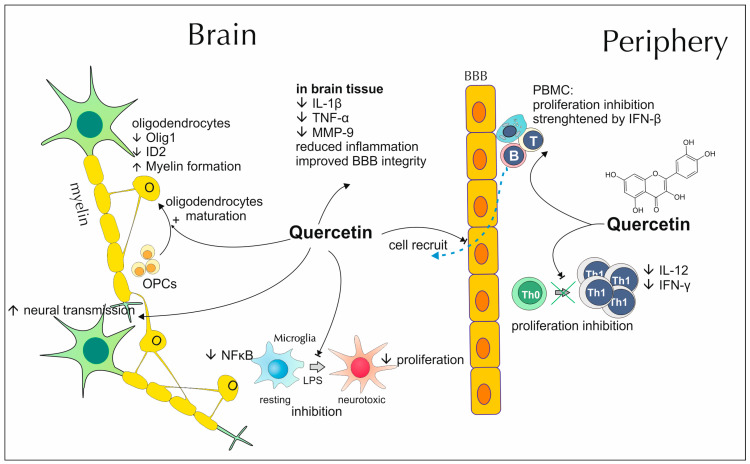
Quercetin’s protective role in demyelination. Quercetin can prevent LPS-driven microglial activation, which leads to neurotoxicity. It supports myelin renewal by activating oligodendrocyte precursor cells (OPCs) and promoting oligodendrocyte maturation. Furthermore, quercetin downregulates the secretion of pro-inflammatory proteins and MMP-9 in brain tissue, reducing inflammation and supporting blood–brain barrier (BBB) integrity. Additionally, in the periphery, quercetin can modulate T cell responses to autoantigens by promoting cell proliferation and inhibiting Th1 cell responses.

In vitro studies suggest the beneficial effects of quercetin on inflammatory processes and the proliferation of cells obtained from persons affected with demyelination (Figure 4). In a study conducted by Sternberg et al., quercetin showed dose-dependent antiproliferative activity on PBMCs isolated from MS-affected donors. This effect was not related to cytotoxic activity and was strengthened by the addition of IFN-β, a drug standardly used in the control of MS clinical symptoms. Similar inhibition was observed in the production of proinflammatory cytokines–IL-1β or TNF-α as also MMP9-, a proteinase that may disrupt BBB as well as directly impair axons [97]. Similar capacities can be observed for chemically modified polyphenol–quercetin pentaacetate, which was a strong inhibitor of IL-17A expression as well [98]. A reduction in cell proliferation rate was also reported for quercetin-treated reactive microglia. Furthermore, the application of this polyphenol contributes to the suppression of the inflammatory NF-κβ signaling pathway and counteracts LPS-induced polarization into neurotoxic microglia phenotype [99]. Mast cells are considered an important element in the regulation of BBB permeability and thus they can contribute to the pathology of many neuroinflammatory illnesses, including MS, by altering the influx of numerous immune system cell fractions or directly penetrating the barrier and releasing proinflammatory compounds. Quercetin serves as a mast cells inhibitor, as it is capable of counteracting their basic and up-regulated secretion of IL-6, an inflammatory cytokine that enhances neuronal dysfunction caused by other cytokines, and tryptase, a protein that promotes expression of endothelial adhesion molecules through stimulation of proteinase-activated receptors (PARs) [100]. The anti-inflammatory and antiproliferative potential of quercetin was also observed in T cells isolated from EAE-affected mice. Incubation with the mentioned flavonol restrains T cell proliferation and secretion of IFN-γ and IL-12 in a dose-dependent manner. These two cytokines are important for the promotion of Th1 cell differentiation, which in turn contributes to EAE and MS pathology [101]. Extract prepared from *Dendropanax morbifera’s* leaves contains high amounts of quercetin. Kim et al. demonstrated that in mice-derived cell cultures of maturing astrocytes, oligodendrocytes and microglia it significantly increases oligodendrocytes development and their membrane sheath creation, accompanied with high MBP production and downregulated expression of Oligodendrocyte transcription factor 1 (Olig1) and DNA-binding protein inhibitor 2 (ID2), typically observed among differentiating, myelin-synthesizing oligodendrocytes. Moreover, in cocultures composed of neurons and oligodendrocytes, it boosts the axonal contact formation [102].

Research conducted by Muthian et al., using the EAE mice model, indicates that quercetin is capable of ameliorating this disease intensity, as rodents treated with mentioned polyphenol develop milder symptoms and they return to their pre-immunization state more quickly. In histological analyses, it is reflected in a less apparent infiltration of inflammatory cells and demyelination of CNS tissues [101]. Similar data towards alleviation of motor impairment comes from a study performed on a rat model of ethidium bromide-caused demyelination [103]. A positive influence, regarding the mitigation of demyelinating processes, was also observed for the lysolecithin-induced focal demyelination model of the optic nerve. Among rats dosed with quercetin, the visual pulse conductivity was higher and less impaired. Furthermore, the ratio of the demyelinated area and the microglia activation, measured in the optic chiasm, were lower [104]. Patients and animals, suffering from demyelinating diseases, present an increased activity of peripheral blood leukocytes’ cholinergic enzymes, exactly butyrylcholinesterase (BuChE) and acetylcholinesterase (AChE). As a result, they experience an impairment in the acetylcholine-mediated inhibition of pro-inflammatory cytokines secretion. Carvalho et al. proved that quercetin may reverse the mentioned effect and restore normal cholinesterase activity in ethidium bromide-induced rat model of demyelination [105].

## 3. Polyphenols’ Role in Treatment of Neurodegenerative Diseases

### 3.1. Overview of Alzheimer’s and Parkinson’s Diseases

Alzheimer’s disease (AD) is one of the most common neurodegenerative pathology concerning mainly people over 65 years of age [106]. AD manifests itself in the loss of cognition ability along with aberrant behaviors in social settings. The pathological hallmarks of this disease are amyloid-β (Aβ) plaques formed into fibrillar aggregates and Tau protein in the shape of neurofibrillary tangles. Their clustering along neurons leads to neurodegeneration. Additionally, the presence of brain neuroinflammation caused by oxidative stress altering the functioning of mitochondria is thought to be the etiological factor of AD [107,108]. In 2016 taking care of 35 million patients cost over $226 billion. Prognoses are indicating that the number of new cases will grow leading to the arising of the new epidemic [106]. Taking into consideration the unfavorable predictions and the fact that currently there is no effective treatment, focusing on alternative forms of therapy such as polyphenols can become beneficial [107].

Parkinson’s disease (PD) is another common neurodegenerative disorder affecting elderly patients, two times more often in men than in women. Loss of dopaminergic neurons in the substantia nigra along with the appearance of Lewy bodies in the affected sites are the characteristic features of PD. The consequences of this process are visible mainly as motor symptoms such as tremors and rigidity of the body along with nonmotor symptoms which are pain and behavior alterations. Similarly to the pathogenesis of AD, the causes of PD are thought to be neuroinflammation and mitochondrial damage which results in the presence of oxidative stress. The exact reason for PD occurrence is not known currently. The utilized treatment is based on the use of levodopa which allows for the production of a higher amount of dopamine and reduces the incidence of symptoms. However, there are some serious side effects such as nausea, hallucinations, and dyskinesia. To avoid those afflictions, focusing on alternative forms of treatment would be advantageous [109,110].

Taking into consideration the highest prevalence of both diseases among all of the neurodegenerative conditions and their relevant impact on socioeconomic aspects, it is worth focusing on seeking ways of their prevention and treatment. AD and PD due to their common presence are the most studied neurodegenerative diseases. The substantial amount of knowledge regarding AD and PD is the reason why the polyphenols’ mode of action in these diseases can be discovered and studied extensively.

### 3.2. The Importance of Resveratrol in Treatment of Alzheimer’s and Parkinson’s Diseases

**Figure 5:**
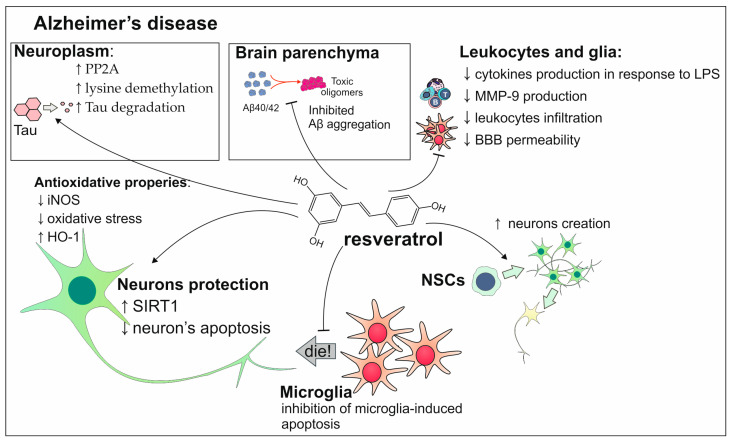
Role of Resveratrol in Alzheimer’s disease. Resveratrol prevents amyloid and tau aggregation in the brain and inhibits neuronal apoptosis through SIRT1 activation while minimizing oxidative stress. Additionally, resveratrol may decrease the inflammatory response in leukocytes and inhibit microglial neurotoxicity.

The involvement of resveratrol in the prevention of AD is based on the fact that this polyphenol also has anti-inflammatory and antioxidant qualities. The way of its action is currently not fully elucidated; however, there are studies suggesting some mechanisms (Figure 5). Resveratrol can inhibit gene expression of LPs-induced pro-inflammatory cytokines and complement proteins. It also enhances apoptosis mediated by microglia. The aggregation of Aβ is enhanced by ROS which leads to increased oxidative stress. The resveratrol’s activity is based on the inhibition of iNOS which results in reduced lipid peroxidation in neurons and by increasing the production of heme oxygenase-1 (HO-1) it minimizes the consequences of oxidative stress. Resveratrol also acts on the degradation of tau protein through phosphatase 2 A (PP2A) activation and demethylation of lysine residues leading to proteasomal degradation [111,112]. In the clinical trial conducted on patients with mild–moderate AD, the intake of resveratrol led to a lowered level of MMP9 in CSF. This can result in decreased BBB permeability which in consequence will reduce the infiltration of leukocytes and therefore inflammation [113]. Resveratrol also has the ability to influence neuronal growth and differentiation. This polyphenol activates SIRT1 which is crucial in these processes and also prevents their apoptosis [114].

**Figure 6:**
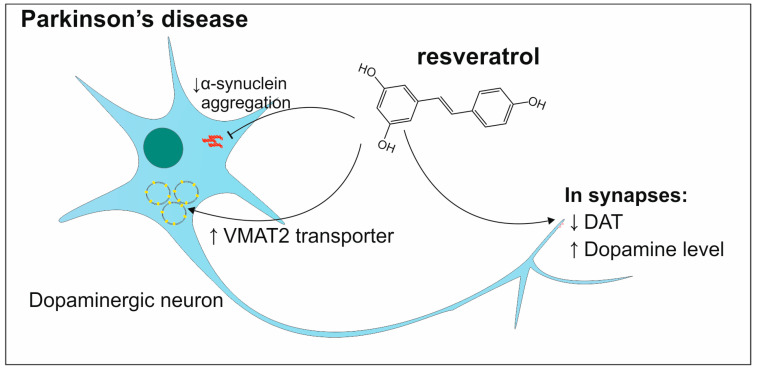
Role of Resveratrol in Parkinson’s disease. Resveratrol may minimize the clinical signs of dopaminergic neuron loss by improving monoamine transport to presynaptic vesicles (elevated VMAT2 levels) and facilitating dopamine exocytotic release. Resveratrol prolongs the presence of dopamine in the synaptic cleft by downregulating DAT and reducing dopamine reuptake. Additionally, resveratrol may decrease α-synuclein aggregation in neurons, thereby preventing neuronal loss.

Resveratrol also turns out to be an effective agent in the prevention and treatment of PD (Figure 6). The comprehensive study conducted by Singh et al. on 6-OHDA rat models of PD shows many positive effects of resveratrol application. This polyphenol has the ability to restore the level of dopamine from 69% in the group without the intervention of 6-OHDA to 85% after resveratrol administration and 6-OHDA administration. The neurotransmission of dopamine was also evaluated where the levels of synaptic proteins dopamine transporter (DAT) and vesicular monoamine transporter 2 (VMAT 2) were measured. The results show that in the groups of rats treated with 6-OHDA and additionally receiving resveratrol in comparison to the groups without this polyphenol, the level of DAT decreased and VMAT 2 was elevated. This indicates that resveratrol has an influence on the regulation of dopamine transport [115]. Resveratrol is also another polyphenol with the property to inhibit the aggregation of α-synuclein [116]. 

### 3.3. The Importance of Curcumin in Treatment of Alzheimer’s and Parkinson’s Diseases

**Figure 7:**
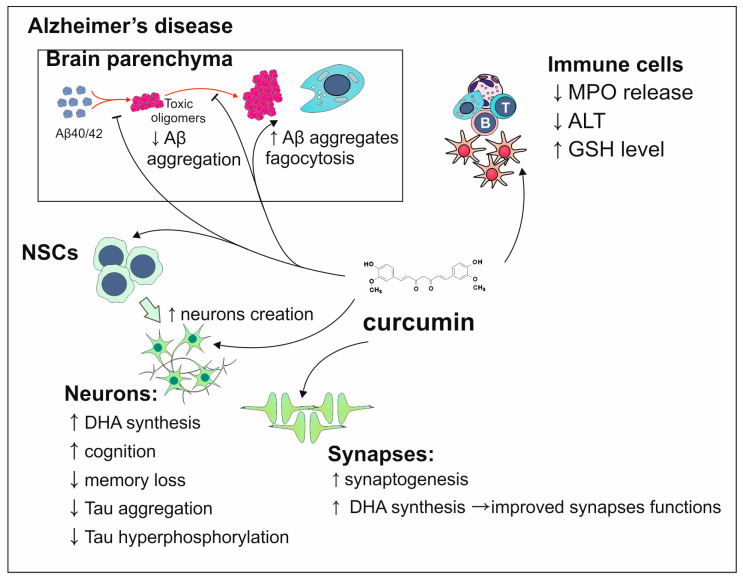
Curcumin’s action in Alzheimer’s disease. Curcumin decreases oxidative stress in immune cells, prevents amyloid β aggregation, and supports aggregate clearance. In neurons, curcumin elevates DHA synthesis, prevents tau aggregation and hyperphosphorylation, stimulates synaptogenesis, and improves synaptic function. Moreover, curcumin activates the differentiation of neuronal stem cells into new neurons, supporting cognitive functions.

The preventive actions taken to avoid chronic diseases such as diabetes and obesity are based on following a diet with high amounts of fruits and vegetables. This approach provides a considerable intake of polyphenols having a positive influence in the prevention of chronic diseases. Studies indicate that curcumin—one of the polyphenols—reduces their morbidity rate which confirms studies where its intake restores insulin levels [106,107]. In the context of AD, the elimination of its risk factors which are chronic diseases significantly reduces the chances for the manifestation [106]. The research conducted on healthy rodents shows that curcumin intake positively impacts their memory by involvement in creating new neurons and synapses [117,118]. The proper functioning of synapses and mitochondria is provided by docosahexaenoic acid synthesis which is enhanced by curcumin. The studies regarding the population of senior healthy people point out that curcumin can lead to the decrease in inflammation by decreasing levels of alanine aminotransferase and plasma myeloperoxidase. The supplementation with curcumin formulated in liposomes led to boosted cognition which in AD requires enhancement [106]. Curcumin is also considered a suitable candidate for being an AD therapeutic due to its ability to bind to Aβ which results in decreased aggregation of amyloid plaques. This polyphenol also inhibits amyloid precursor proteins (APP) metabolism and supports macrophages in Aβ absorption. Considering Tau protein, studies indicate that curcumin inhibits its hyperphosphorylation and aggregation. In the context of restricting oxidative stress, curcumin also plays a role by increasing the amount of glutathione leading to the inhibition of 3-nitrotyrosine production whose presence indicates inflammation [119,120]. Summarized modes of curcumin’s action on processes related to AD development have been shown in Figure 7.

**Figure 8:**
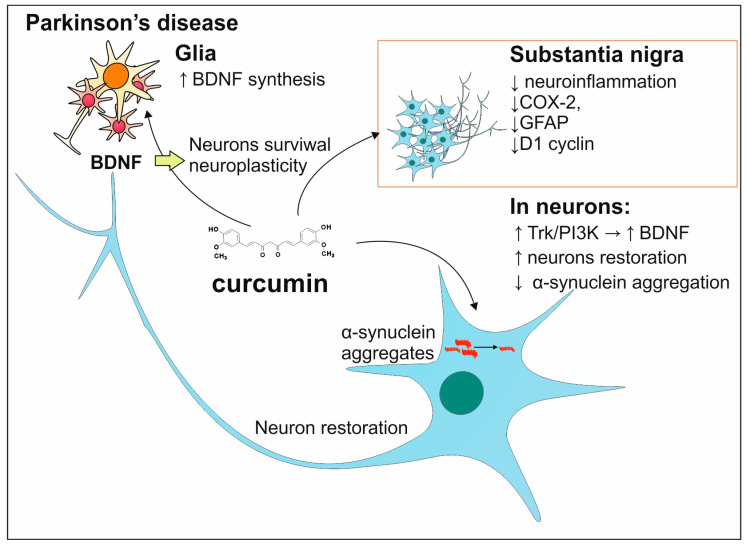
Curcumin’s action in Parkinson’s disease. Curcumin decreases α-synuclein aggregation in neurons and stimulates Trk/PI3K-dependent BDNF synthesis in neurons and glial cells, which in turn increases neuronal survival and restoration. Curcumin prevents the loss of dopaminergic neurons in the substantia nigra by decreasing neuroinflammation and inhibiting the activation of neurotoxic astrocytes.

In the case of PD, the incidence of this disease was observed less frequently in the population of Southeastern Asia in comparison to Caucasians. The differences can be explained by the various types of diets in both groups. The cuisine of India is known for the common usage of turmeric which is the source of curcumin having a positive influence on the prevention and treatment of PD. This antioxidant prevents the harmful activity of ROS and toxic metals leading to neurodegeneration which in the context of PD protects dopaminergic neurons [121]. The study conducted on the 6-hydroxydopamine (6-OHDA) PD animal model shows that curcumin influences the Trk/PI3K pathway in a way that the amount of brain-derived neurotrophic factor (BDNF) increases and leads to the restoration of neurons. Curcumin also counteracts neuroinflammation in substantia nigra by suppressing the production of pro-inflammatory agents such as cycloxygenase-2 (COX-2), glial fibrillary acidic protein (GFAP) level, and cyclin D1 [122]. Additionally, curcumin prevents the aggregation of α-synuclein which is the main component of the Lewy bodies [123]. The effects of curcumin on processes associated with PD have been shown in Figure 8.

Despite the promising effects of curcumin, the clinical trials conducted with its use indicated that oral intake results in poor absorption and effectiveness [120,124]. The various approaches are tested to deliver curcumin and exploit its potential. Into consideration are phospholipids complexes, emulsion-based delivery systems, liposomes, polymeric micelles and curcumin nanoparticles [125].

### 3.4. The Importance of Luteolin in Treatment of Alzheimer’s and Parkinson’s Diseases

**Figure 9:**
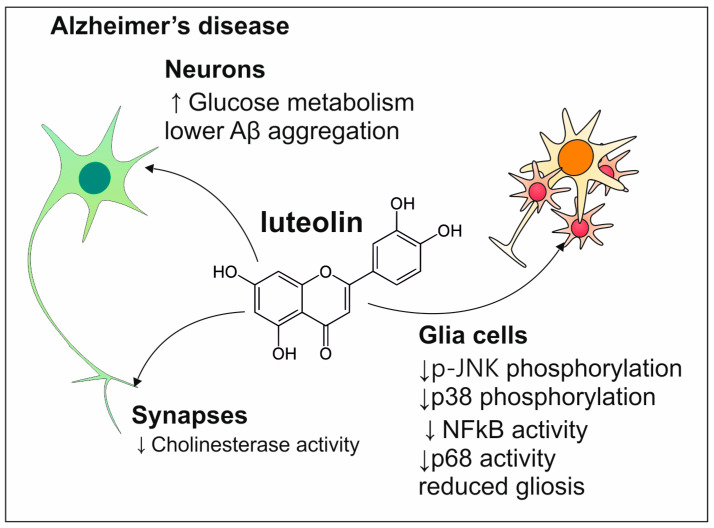
Reported actions of luteolin on Alzheimer’s disease. Luteolin may reduce the activation of glial cells by decreasing p-JNK and p38 phosphorylation as well as NFκB activity. In neurons, luteolin increases glucose uptake and metabolism and decreases Aβ aggregation. Luteolin has also been shown to enhance cholinergic transmission by regulating cholinesterase activity.

Luteolin exhibits properties that are similar to other representatives of polyphenols. The studies in vitro and in vivo show that luteolin acts in a neuroprotective way which is why this polyphenol would be the appropriate option for the prevention and treatment of AD (Figure 9). In the study conducted on mice, it was proved that on the molecular level, luteolin has the ability to inhibit phosphorylation of MAP kinases such as p-JNK and p38 which are responsible for the induction of gliosis, and release of inflammatory mediators (p-NF-kB and p65) leading to neuroinflammation and in consequence to neurodegeneration [126]. The anti-inflammatory quality of luteolin is also expressed in mast cell inhibition. Luteolin as the structural analog of quercetin also acts as an AChE inhibitor [124]. The ability of luteolin to enhance the metabolism of glucose contributes to the suppression of Aβ aggregation because it is increased when the brain is resistant to insulin [127].

**Figure 10:**
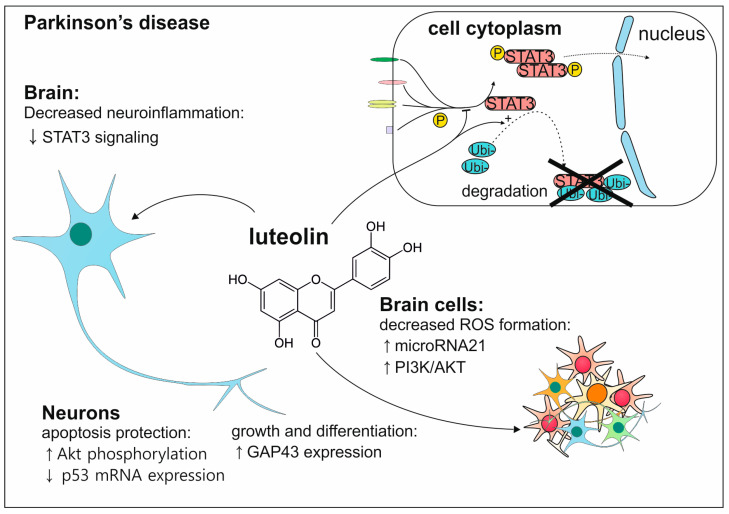
Luteolin’s activity in Parkinson’s disease. Luteolin has been shown to inhibit STAT3 signaling by interfering with its phosphorylation and promoting ubiquitin-dependent degradation. Additionally, luteolin may prevent neuronal apoptosis by downregulating p53, increasing AKT phosphorylation and regulating ROS formation.

Luteolin can also act in several ways to maintain the proper condition of dopaminergic neurons leading to the prevention of PD (Figure 10). Firstly, luteolin causes a reduction in neuroinflammation by activation of STAT3 and by prevention of its activated version migrating to the nucleus. Secondly, the reduction in ROS by luteolin is obtained by increased expression of microRNA21 leading to activation of the P13k/Akt pathway. Additionally, neurons are stimulated to growth by enhancement of neurite outgrowth, and by increase in growth associated protein 43 (GAP43) expression, they are able to differentiate. Neurons have also a higher chance of survival thanks to the ability of luteolin to prevent apoptosis in many ways i.e., by increasing Akt phosphorylation and P53 mRNA [128].

### 3.5. The Importance of Quercetin in Treatment of Alzheimer’s and Parkinson’s Diseases

**Figure 11:**
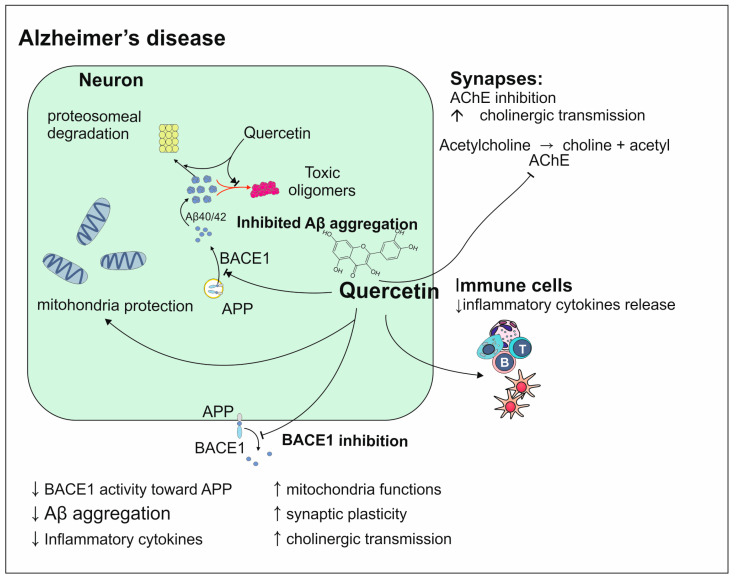
Role of Quercetin in Alzheimer’s disease. Quercetin inhibits APP cleavage by BACE1 and prevents Aβ aggregation into toxic oligomers. It also promotes the proteasomal degradation of Aβ40/42. Additionally, quercetin improves mitochondrial function, cholinergic transmission and synaptic plasticity. Quercetin can decrease neuroinflammation by downregulating the release of inflammatory cytokines from immune cells.

Quercetin acts similarly to the previously mentioned polyphenols in the prevention and treatment of AD. Quercetin is thought to be one of the most effective polyphenols. Its way of action is based on antioxidant and anti-inflammatory qualities (Figure 11). The study conducted on mice models of AD showed that the use of quercetin in the long term leads to improvement of mitochondria functioning [129]. Many studies on neurodegenerative diseases proved its neuroprotective activity. The research performed on *Caenorhabditis elegans* provides data indicating that quercetin removes pathological changes characteristic of AD and has a positive influence on synaptic plasticity [129]. Similarly to other polyphenols, quercetin prevents Aβ aggregation through its degradation in proteasomes and by suppressing the activity of beta-site APP cleaving enzyme 1 (BACE1) protease that inappropriately cuts APP. The ability of quercetin to maintain neurons in the proper condition is fulfilled mainly by influencing the production of cytokines. This action can take place thanks to quercetin’s impact on cellular pathways such as PI3K/Akt, Nrf2 pathway, and mitogen-activated protein kinase (MAPK) signaling cascades [130]. The reduced number of cholinergic neurons and choline acetyltransferase (ChAT) is often detected in AD and it is suggested that the lower level of the neurotransmitter which is acetylcholine is one of the reasons for worsened cognition. The studies indicate that quercetin acts as an acetylcholine esterase inhibitor which means that the cholinergic transmission is not interrupted [131]. However, despite all of the advantages of quercetin, its potential cannot be fully exploited because of poor bioavailability [129].

**Figure 12:**
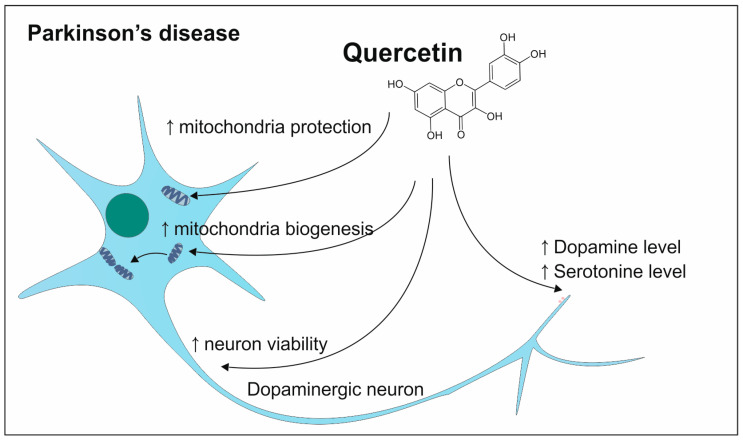
Quercetin’s role in Parkinson’s disease. Quercetin improves the function of dopaminergic neurons by promoting mitochondrial biogenesis and enhancing neuronal viability. It also increases the release of dopaminergic neurotransmitters.

Quercetin also can have an application in the prevention and treatment of PD (Figure 12). The study conducted on rats with implemented 6-OHDA shows that quercetin received for 14 days long led to an increased amount of dopamine and 5-hydroxytryptamine (5HT). Quercetin intake also resulted in a higher number of preserved living neurons. Another study confirms the efficacy of the high doses of quercetin providing evidence of the arrested tremor in rats [132,133]. This polyphenol also takes part in the preventive actions protecting the mitochondria of neurons from damage. The studies on animal models show the versatility of quercetin in its way of action. This polyphenol can inhibit the decline of mitochondrial complex-I activity, and it also leads to increased biogenesis of mitochondria [134]. In the context of the prevention of mitochondria from ROS activity, the research on the rotenone-administered animal model provides evidence of increasing the activity of enzymes acting antioxidative [133].

## 4. Polyphenols’ Role in Treatment of Epilepsy

Epilepsy affects over 70 million people all around the world, and the prevalence is highest in groups of infants and older adults [135]. The disorder is a symptom complex with multiple risk factors and a strong genetic predisposition rather than a condition with a single expression and cause. According to the International League Against Epilepsy (ILAE), the diagnosis of epilepsy requires at least two unprovoked epileptic seizures or a single unprovoked seizure with a risk of recurrence ≥60% over ten years [136]. The causes are divided into six categories: genetic, structural, metabolic, infectious, immune and unknown. Although the natural history of untreated epilepsy remains unclear, its symptoms reliably decrease patients’ quality of life and can potentially be life-threatening. Premature deaths can be directly attributable to epilepsy by status epilepticus, injuries, sudden unexpected death of epilepsy (SUDEP), or indirectly by aspiration pneumonia, suicide, drowning or car accident. Etiology, comorbidity and abnormalities in the electroencephalogram, type of seizures and the number of seizures experienced before treatment onset are all considered prognostic factors [135]. The prognosis is also related to the likelihood of attaining seizure freedom on treatment [135,137]. Pathophysiology of epileptogenesis comprises the process of converting a non-epileptic brain into one capable of generating spontaneous, recurrent seizures, leading to an imbalance between excitatory and inhibitory activity within a neuronal network. The brain pathological findings include synaptic reorganization, glial function and structure alterations, and loss of excitatory and inhibitory neurons in specific subfields such as the hippocampus. Antibodies targeting glutamic acid decarboxylase 65 (GAD-65), leucine-rich glioma inactivated 1 (LGI1), contactin-associated protein-like 2 (CASPR2) and N-methyl-D-aspartate (NMDA) receptors are the most frequent basis of autoimmune epilepsy. Potential causes of seizures or aggravation of epileptic symptoms are pro-inflammatory processes (such as IL-1β, TGF- β and activin receptor-like kinase), changes in ion channels and dysregulation of intracellular signaling cascades (such as BDNF and tropomyosin receptor kinase, the mechanistic target of the rapamycin (mTOR) pathway, adenosine/adenosine kinase and microglia activation) [135]. These causes are possible targets for antiepileptogenic or disease-modifying therapies, which can include polyphenols (e.g., resveratrol, curcumin, luteolin and quercetin). Current therapeutic strategies are either pharmacological or non-pharmacological. Oral antiepileptic drugs (AEDs) are the first-line treatment right after diagnosis. They include old-generation medications—e.g., valproic acid, phenytoin or carbamazepine and new-generation ones such as lamotrigine, topiramate and gabapentin. Oral intake of AEDs is associated with numerous side effects and implies numerous drug interactions in patients with comorbidities [136]. Non-pharmacological approaches include palliative methods—surgery and neuromodulation. Epilepsy surgery encompasses curative procedures, such as corpus callosotomy and implantation of stimulation devices. In pre-surgical evaluation epileptogenic zones are identified, to prevent postoperative neurological and cognitive deficits. Neuromodulation methods comprise vagus nerve stimulation (VNS), deep brain stimulation of the anterior nucleus of the thalamus (ANT-DBS) and closed-loop responsive neurostimulation of the epileptogenic zone or zones. These methods should be considered for patients with epilepsy in whom two or three anti-epileptic medications have been ineffective [138,139]. Polyphenols, especially flavonoids, are known for their antioxidant, hypocholesterolemic, and anti-inflammatory effects and ability to modulate cell signaling, which plays a crucial role in epileptogenesis [140]. Contrary to AEDs, their intake does not correlate with interfered cognitive functions. Moreover, their neuroprotective effects improve cognitive performance induced by stress factors [141]. Flavonoids’ possible role in attenuating adverse effects of AEDs, such as nephrotoxicity, and their own anti-epileptic properties, make them promising adjuvant medicaments for epilepsy treatment by enhancing multiple anti-epileptic mechanisms [142,143,144].

### 4.1. Resveratrol’s Role in Treatment of Epilepsy

Studies in mice and adult zebrafish demonstrate that resveratrol interacts with a broad spectrum of targets as a potential antiepileptogenic agent. It is also vital for its neuroprotective and antioxidant effects, which are critical contributors to epileptogenesis. Mice treated with a ten-day course of resveratrol after pentylenetetrazole (PTZ)-induced chronic epilepsy developed milder seizures than untreated mice, and the latency of seizures was longer. Resveratrol had a protective effect in rat hippocampal (CA)-1 and CA-3 neurons compared with the model groups that did not receive resveratrol in histological examination. Moreover, it significantly lowered the calcium-binding protein B (S100B) in serum and CSF, which suggests improved blood–brain barrier permeability and lowered astrocytes loss and brain damage. [145]. Zamora-Bello et al.‘s study obtained convergent results in increasing latency and decreasing severity of seizures, suggesting resveratrol can play a role as an adjuvant in treating epilepsy [146]. In line with previous studies, Wu et al. performed behavior monitoring, intracranial electroencephalography recording, histological analysis and Western blotting to evaluate the anti-epilepsy effect of resveratrol in kainate-induced epileptic rats [147]. Primary glutamate receptors responsible for the development of seizures are NMDA receptors, α-amino-3-hydroxy-5-methyl-4-isoxazolepropionic acid (AMPA) receptors and kainate receptors (KARs), especially Glu R6. Overexpression of KARs promotes seizure development [147]. The study confirmed a reduction in the frequency of spontaneous seizures, inhibited the epileptiform discharges and protected CA1 and CA3 hippocampal region neurons from kainate-acid-induced death. It showed the preservation of KARs in the hippocampus, which might be an underlying mechanism for the anti-epileptic function of resveratrol . Emphasizing the hippocampus as the key region for developing status epilepticus, a study by Chuang et al. examined the neuroprotective effect of resveratrol on mitochondrial biogenesis in the hippocampus following experimental status epilepticus, performing measurements of nuclear respiratory factor 1 (NRF1), mitochondrial transcription factor A (Tfam), COX and mtDNA amounts for evaluation of mitochondrial biogenesis, resulting in up-regulation of all following parameters apart from reduced activated caspase-3 activity and attenuated neuronal cell damage [148]. It highlighted that resveratrol plays a pivotal role in the mitochondrial biogenesis machinery that may provide a protective mechanism counteracting seizure-induced neuronal damage by activating the peroxisome proliferator-activated receptor-γ coactivator 1α (PGC-1α) signaling pathway [148]. Effects of resveratrol on seizures and hyperexcitable neuronal activity associated with the activation of NMDA receptors and inhibition of voltage-gated potassium channels are evaluated in the study by Wang et al. In their work, resveratrol was not suitable for the seizures caused by disturbance of the voltage-dependent potassium channels, but more importantly, it increased the NMDA-induced seizure thresholds and suppressed the frequency of NMDA/glycine-evoked excitatory field potentials (EFPs) and action potentials. Resveratrol not only lowered the 4-aminopyridine (4-AP)-induced thresholds for myoclonic twitch and face and forelimb clonus but enhanced the thresholds for running and bouncing clonus and tonic hindlimb extension at the higher dose (50 mg/kg) [149]. Comparable results, obtained for administration of resveratrol an hour after status epilepticus onset, included protection of glutamatergic hippocampal regions from cell death, diminishing loss of gamma-aminobutyric acid (GABA)-ergic interneurons, oxidative stress easing and a decrease in TNF-α concentration with no behavioral changes in comparison diazepam, in a study by Mishra et al. [150]. Li et al. analyzed the mechanism underlying the potential anti-epileptic effect of resveratrol, focusing on the expression of kainate receptor subunit 2 (GluK2) and—gamma-aminobutyric acid receptor α1 GABAR-α1 in various phases of status epilepticus and its influence on the ratio of Glu to GABA in the hippocampus with kainate acid rats’ brain injections. It showed that intragastrical administration of resveratrol in the chronic phase down-regulated the expression of hippocampal kainate glutamate receptor–GluK2 and upregulated the GABAR-α1 subunit expression, which is reverse to epileptic mechanism and induces anti-epileptic effect. Acute resveratrol perfusion in a dose of 50 μmol/L upregulated the expression of GluK2 and GABAR-a1 and reduced epileptiform discharges in the CA1 hippocampal area. Also, resveratrol increased the expression of GABAR-α1 in the silent phase [151]. A study from 2022 exploring the influence of intraperitoneal injection of a single dose of resveratrol on adult zebrafish and zebrafish larvae did not show anticonvulsant properties in the PTZ-induced seizure model regarding neuroprotective and anti-epileptogenic properties. The expression level of genes related to neuronal activity (c-fos), inflammatory response (IL-1β), neurogenesis (p70S6Ka and p70S6Kb) and apoptosis (caspase-3) 24 h after PTZ exposure showed no differences with control probe, which indicated that way of administration was insufficient [152]. However, clinical resveratrol use is impaired by its poor absorption, low bioavailability and rapid metabolism, resulting in a deficient brain distribution. Ways to deliver resveratrol to the brain to make it more effective were studied. Due to supercritical fluid micronization technology (SEDS), Almeida showed that pretreatment with micronized resveratrol decreased the occurrence of the tonic-clonic seizure stage III of Racine’s classification and slowed the development of the PTZ-induced seizures in a similar manner of diazepam. While non-processed resveratrol was not able to protect the animals against PTZ-induced seizures, it did not change the occurrence or the latency of seizure development. Also, the rise in c-fos transcript levels was prevented by micronized resveratrol. Additionally, micronized resveratrol prevented behavioral alterations induced by seizures [153]. Similar results were acquired by Ethemoglu et al. They acquired an amphipathic liposomal delivery system, which allowed them to cross the blood–brain barrier better. They reported that although resveratrol (clear) did not significantly change the frequency of the electrocorticogram (ECoG) spikes, the micronized resveratrol lowered the spikes’ frequency and amplitude, indicating its anti-epileptic influence. The study also found that resveratrol significantly lifts levels of antioxidative components like glutathione and s-transferase in hippocampal tissue, leading to reduced epileptic seizures [154]. Other findings, addressing the better bioavailability of resveratrol, suggested that encapsulating resveratrol in glutathione-coated collagen nanoparticles enhances the anti-epileptic potential of resveratrol by delivering it to the brain’s targeted location. Hence, nano resveratrol was more effective in controlling epilepsy seizures than the dose of 1/10th and 1/100th of standard resveratrol and comparable to the dose of 1/1000th of resveratrol, the mean latency time in developing myoclonic jerk and generalized seizure were more prolonged than that of resveratrol and diazepam and did not cause any behavioral change in mice. Improving the biochemical antioxidative markers also marks higher doses of nano resveratrol. Nanoresveratrol, as well as resveratrol, reduced high mobility group box 1 protein HMGB1 and TLR-4 levels, which are inflammatory markers contributing to epileptogenesis. The NF-κβ and COX-2 expressions were significantly upregulated in PTZ-induced mice; however, they were significantly reduced when treated with nano resveratrol [155]. TLR3 plays an essential role in the development of epilepsy after brain insults because it causes a long-lasting increase in seizure susceptibility in rats [156]. Shor et al. studied resveratrol’s impact on pilocarpine-induced epilepsy models, ensuring that resveratrol decreased the frequency of spontaneous recurrent seizures recorded via electroencephalography (EEG) examination. It may be connected to the reduction in TLR3-mediated neuroinflammation in epileptogenesis [157]. Results from studies investigating the effect of resveratrol on epilepsy are summarized in Table 1.

### 4.2. Curcumin’s Role in Treatment of Epilepsy

Curcumin on-treatment as an anti-inflammatory compound in epilepsy and its possible impact on the mTOR pathway was explored on hippocampus brain slices, where curcumin-treated slices revealed suppressed spontaneous seizure activity and lower inflammatory markers (IL-1β and IL-6) [158]. However, there was no inhibition of the mTOR pathway, suggesting that other signaling pathways are involved in curcumin’s anti-epileptic impact. In advance, curcumin seems to affect the MAPK(ERK1/2) signaling pathway, which modulated transcription of the genes involved in the pro-inflammatory processes [158,159].

Curcumin has considerable potential for the control of seizures and cell damage induced by epileptic fits, thereby increasing the therapeutic options available to the patients. It has various neuroprotective properties, targeting many pathways. Curcumin treatment reduced post-seizure increased expression of IL-1 and NLRP3 in rat models of kainate acid-induced epilepsy, concluding that curcumin inhibits KA-induced epileptic syndromes via the suppression of NLRP3 inflammasome activation [160]. Zhu et al. suggest that the L-arginine–nitric oxide pathway may be involved in the anti-epileptic properties of curcumin and that the role of nNOS is prominent in this neuroprotective feature because a chronic administration of curcumin can exert dose-dependent protection against seizures [161]. Another neuroprotective value of curcumin is the reduction in seizure-caused brain damage. Curcumin treatment downregulates TNF-α expression levels and upregulates klotho protein and erythropoietin (EPO) in the mice hippocampus, alleviating neuronal cell death in epileptic brains [162]. Curcumin also attenuates increased expression of astrocyte (GFAP) and microglial (Iba-1) activation markers, preventing cognitive deficits. Moreover, immunohistochemical analysis in a study by Kaur et al. showed a significant reduction in activated glial cells on curcumin administration to PTZ-induced animals [163]. Curcumin’s prominent antioxidative effects can achieve a significant decrease in the frequency of epileptic episodes and an increase in the latency of status epilepticus, as in the study by Ahmad. In his study, pre-treatment with curcumin significantly and dose-dependently attenuated lithium-induced depletion in GSH and increment in lipid peroxidation content (TBARS) compared to lithium only. Not only can the GSH levels be restored by curcumin activity, but there is also a notable reduction in ROS generation, lipid peroxidation and protein carbonyls [164]. Moreover, curcumin can reduce cell loss and restore the activity of mitochondrial complexes to protect mitochondria from seizure-induced structural alterations [165]. In pilocarpine-treated rats, curcumin achieved improvement in hippocampal levels of further oxidative stress exponents–malondialdehyde, nitric oxide and depletion in the activity of Na^+^K^+^-adenosine triphosphatase (ATPase), CAT and AChE [166]. Also, no post-seizure behavioral manifestations were observed after the curcumin treatment of epileptic animals. These results suggest that curcumin supplementation can prevent mitochondrial dysfunctions and oxidative stress with improved cognitive functions in a chronic epilepsy model. Similar promising results were obtained in kainate-acid epilepsy-modelled rats [167]. A study conducted by Jyoti et al. confirmed that high-dose curcumin treatment significantly inhibited the onset of grade III and IV seizures in iron-induced epilepsy in rodents [168]. Here, the antioxidative effects of curcumin, reliable for reduction in lipid peroxidation, may be one of the reasons for the significant inhibition of disruption of Na^+^/K^+^-ATPase activity [168]. Furthermore, Wang et al. found that curcumin protects neuronal cells against status-epilepticus-induced hippocampal neuronal damage in the lithium-pilocarpine-induced status epilepticus rat model through induction of autophagy and inhibition of necroptosis, alteration in expression of Beclin-1 and LC3 proteins for autophagy, and mixed lineage kinase domain-like pseudokinase (MLKL) and serine/threonine-protein kinase 1 (RIP-1) proteins for necroptosis of the post-epileptic rat hippocampus in CA1, CA3, DG and H regions [169]. In the iron-induced epilepsy model, upregulation of epilepsy-associated ion–channel proteins: calcium voltage-gated channel subunit α1 CACNA1A and gamma-aminobutyric acid receptor subunit δ (GABRD) was alleviated by orally administered curcumin supplementation [170]. Also, Kumar et al. observed reduced mRNA and protein levels of upregulated Nav1.1 voltage-gated sodium channels in an exact model [171]. Another aspect of curcumin’s action is modifying the epileptogenesis process. Acute administration of curcumin can significantly increase the seizure threshold in the increasing current electroshock (ICES) model, while chronic administration can prevent post-ictal mortality in rats [172]. Continual administration of curcumin during the latent period of epileptogenesis in the kainite acid (KA)-induced rat temporal lobe epilepsy model resulted in attenuation of the severity of spontaneous recurrent seizures and protected against cognitive impairment. However, it did not prevent the occurrence of spontaneous recurrent seizures [173]. Curcumin can significantly attenuate seizure severity, depression-like behavior and memory impairment in a dose-dependent manner under repeated administration by modulating brain monoamines activity, nitrosative stress levels and acetylcholinesterase activity [174]. The effect of post-pilocarpine amino acids neurotransmitter alterations, such as increased aspartate, glutamate, GABA, glycine and taurine levels, providing a state of hyperexcitability in the hippocampus, reflects one of the epilepsy mechanisms. The administration of curcumin can normalize these impaired intermolecular balances. Moreover, curcumin induces a metabolic shift from the major excitatory to the major inhibitory amino acid, reducing the state of hyperexcitability through the augmentation of GABAergic transmission. In the mentioned research, curcumin treatment resulted in no seizure manifestations in studied animals [175]. Curcumin also plays a role in developing the cognitive deficits associated with epileptogenesis in rats, which may bind with modulating BDNF and its downstream effectors and increased acetylcholinesterase activity. The results indicate the multiple targets of curcumin’s action in its neuroprotective potential [168]. Researchers aimed to increase curcumin’s bioavailability and the ability to cross the blood–brain barrier to improve its promising anti-convulsant abilities and facilitate its potential development as an anti-epileptic drug [176]. One of the methods was developing a curcumin hydroxypropyl-b-cyclodextrin inclusion complex (CURHP-b-CD), resulting in HP-b-CD inclusion complex bioavailability 3.8 times higher that of free curcumin, which caused a more substantial anti-epileptic effect in animals, inhibiting PTZ-induced seizures in zebrafish and mice [177]. Another approach was encapsulating curcumin in solid lipid nanoparticles (SLNs). SLNs reduced neuronal apoptosis through the Bcl-2 family and P38 MAPK pathways. Moreover, it reduced hippocampal neuronal loss and inhibited neuronal apoptosis in the hippocampus of KA-induced rats. The obtained results were better than in non-encapsulated curcumin trials, highlighting pure curcumin particles’ poor and unstable bioavailability [178]. A different study focusing on micronized curcumin showed that the occurrence of the tonic-clonic seizure stage was reduced similarly to valproate, whilst non-micronized curcumin did not achieve equivalent results [179]. Liposome-entrapped curcumin dose-dependently increased the latency and decreased the duration of PTZ-induced clonic seizures during status epilepticus in a study by Agarwal et al. It also caused a significant increase in seizure threshold current and latency to myoclonic and generalized seizures in the ICES test and PTZ-induced seizures, respectively [180]. However, the study presenting similar seizure attenuation by curcumin tried combined therapy with curcumin and diazepam, which resulted in promising results. Combined treatment decreased the seizure threshold and prevented the behavioral progression of the seizure while also reducing the neuronal damage it causes (better than diazepam only) [144]. The above results point out the use of curcumin as an adjuvant agent for epilepsy treatment.

Curcumin in a more bioavailable form (nano curcumin) was administered as an add-on therapy for four weeks to pediatric epileptic patients not responding to medicaments (known as untreatable epilepsy) in a double-blinded randomized crossover clinical trial. Daily 4 mg/kg dose of curcumin was administered orally to patients for four weeks straight, and after a washout period, the number of seizures and changes in inflammatory cytokines, including IL-1α, IL-1β, IL-2, IL-4, L-6, IL-8, IL-10, CCL-2, TNF-α, epidermal growth factor (EGF) and IFN-γ were evaluated. Only two of the mentioned inflammatory cytokines were significantly lowered by curcumin—CCL-2 and TNF-α. However, curcumin significantly reduced the number of seizures at the end of the intervention, which may suggest that curcumin treatment can help treat patients with intractable pediatric epilepsy. Nonetheless, no further research has been performed yet [181]. Results from studies investigating the role of curcumin in epilepsy are summarized in Table 2.

### 4.3. Luteolin’s Role in Treatment of Epilepsy

Epileptic seizures cause an inflammatory response, leading to significant damage in the hippocampus region, associated with heightened levels of proinflammatory molecules, such as nitric dioxide and MMP2. As a reliable anti-oxidative and neuroprotective agent, luteolin decreases MMP2 and iNOS levels with an elevation of endothelial nitric oxide synthase (eNOS), indicating that it has an indirect antioxidant effect [182]. Activation of immune-inflammatory response pathways after an epileptic attack, such as the TLR4/IκBα/NF-κB pathway, can be alleviated by luteolin. Luteolin decreases the expression of TLR4, reduces phosphorylation levels of IκBα and NF-κB and decreases levels of cytokines TNF-α, IL-6, IL-1β and IL-10 in PTZ-induced rats’ brains. Moreover, luteolin can restore post-seizure increased malondialdehyde and depleted GSH levels [183]. Similar results were obtained by Cheng et al. The impact of luteolin in epileptic seizures in the mentioned studies was expressed by significantly reduced seizure intensity and duration and prolonged latency period of seizures. Also, behavioral deficits and cognitive impairment have improved. The results suggest that luteolin can be a nutrient supplement for epileptic patients [184]. However, in the study by Tambe et al., luteolin at all doses very efficiently protected the animals from PTZ-induced seizures. Seizure-induced cognitive impairment is exerted by suppression of ROS production and activation of PKA/CREB/BDNF signaling in the hippocampus. BDNF-mediated increase in synaptic plasticity and neuronal resistance against epilepsy-associated damage may inhibit oxidative stress and lead to cognitive improvement. Luteolin treatment also promotes the inhibition of the mTOR/4E-BP1 signaling pathway, which is involved in epileptogenesis, in PC12 neuronal cells [185,186]. Luteolin exhibits a protective effect against excitotoxic brain injuries by significantly reducing post-seizure hippocampal glutamate levels. Pretreatment with luteolin beneficially prevents glutamate-mediated overexcitation in the generation of KA-induced seizures. Lin et al. suggest that the neuroprotective effect of luteolin against KA-induced hippocampal neuronal damage is attributable to its anti-inflammatory effect, and it makes luteolin a promising candidate for preventing and treating glutamate excitotoxicity-related brain disorders [187]. Inborn severe neurodevelopmental disorder caused by mutations in the X-linked cyclin-dependent kinase-like 5 (CDKL5) gene, CDKL5 deficiency disorder (CDD) includes symptomatology such as early infantile onset refractory epilepsy. In CDD, chronic luteolin treatment has a therapeutic value because it can recover microglia overactivation, hippocampal neurogenesis, dendritic pathology, and behavioral abnormalities. The underlying mechanisms can compound luteolin’s neuroprotective effects, such as increased BDNF expression in the cerebral cortex. Nevertheless, primarily, luteolin increases the rate of cell proliferation in the sub-granular zone of the hippocampal dentate gyrus of Cdkl5 +/− mice and promotes the restoration of the balance between mature and immature spines in the hippocampus and cortex and postsynaptic density protein-95 (PSD-95), a crucial excitatory postsynaptic scaffold protein required for synaptic stabilization [188]. A similar study by Galvani et al. showed restored microglia body size and shape and, significantly, proinflammatory cytokines and P-STAT3 levels in CDD rats after luteolin treatment. Moreover, pretreatment with luteolin partially restored NMDA-induced tonic-clonic seizure persistence, underlying its inhibition of proinflammatory cytokines excreted by activated microglia cells [189]. Systematic evaluation of luteolin’s effects in anti-epileptic therapy concluded that luteolin suppressed seizure induction, duration and severity following PTZ injection, reversed cognitive impairment, reduced neuronal and oxidative stress damage and increased phosphoactivation of protein kinase A (PKA) and cAMP-response element binding protein (CREB) as well as BDNF expression. However, the authors suggest that this does not exclude other possible luteolin-related anti-epileptic mechanisms [141]. The use of luteolin in the treatment of epilepsy has its limitations–the rate of absorption and availability of luteolin is low due to its complex structure and large molecular weight [140]. However, a microemulsion system can improve luteolin’s water dispersibility and bioavailability [190]. An in vitro study comparing the effects of luteolin and micronized luteolin on the severity and latency of seizure incidence did not demonstrate either substance’s efficacy. Moreover, this study’s limitation was the inability to examine luteolin penetration into the brain cells of the test organisms [191]. Results from studies on the use of luteolin in epilepsy models are summarized in Table 3.

### 4.4. Quercetin’s Role and Treatment of Epilepsy

The exact mechanism of quercetin’s effect on epilepsy is unidentified. Potential underlying mechanisms reliable for its anti-epileptogenic effect are interactions with GABA, acetylcholine, glycine, adenosine and serotonin receptors and its neuroprotective and anti-inflammatory properties [143,192]. According to Carmona-Aparicio et al., epileptic seizures are usually positively correlated with the levels of oxidative stress markers. Quercetin lowered oxidative stress markers, decreasing the severity and increasing the latency of seizures. Their study suggests that antioxidant and anti-epileptic properties were modulated via direct scavenging of ROS and antioxidant enzyme activity [193]. Align with downregulation of the pro-inflammatory cytokines such as TNF-α, IL-1β and IL-6 and upregulation of the anti-inflammatory cytokines—IL-1Ra, IL-4 and IL-10, quercetin has a high binding affinity with the synaptic vesicle 2A (SV2A) protein, which modulates exocytosis of the transmitter-containing vesicle. The binding affinity of quercetin is comparable to the standard anti-epileptic drug—levetiracetam [194]. Quercetin also attenuates the production of proinflammatory cytokines such as TNF-α and IL-1β. It suppresses NF-κβ activation and ionized calcium-binding adapter molecule 1 in microglia cells [143]. In the study by Nassiri-Asl et al., alleviation of the severity stage of the seizure, as well as a significant increase in the step-through latency of the passive avoidance response, was achieved by intraperitoneal administration of quercetin in small doses before inducing an epileptic state. Authors suggest that quercetin modulates GABA receptors, as it has been reported for other flavonoids [195]. Modulation of the expression of the GABA receptor β1 and β3 by quercetin was also demonstrated by Moghbelinejad et al. They suggest that it may prevent any progress in neurodegenerative and compensatory responses [196]. Blockade of NMDA receptors decreases brain damage related to epilepsy, which makes it a considerable drug target. Quercetin shows a high-affinity interaction with NMDAR and significantly delays the onset of seizures in pilocarpine-induced seizure models [197]. Quercetin’s ability to cross the blood–brain barrier and induce apoptosis in senescent cells may be essential in the clinical management of mTOR-related epilepsy, such as pediatric drug-resistant epilepsy linked with focal cortical dysplasia type II. Treatment combined with a pan tyrosine kinase inhibitor, dasatinib, significantly reduces the number of neurons against pS6 protein (a standard readout for mTOR activity) and seizure frequency [198]. Evaluating the bioavailability of quercetin may improve its anti-epileptic properties, and lower doses may be required. Hashemian et al. described a novel form of quercetin-conjugated Fe_3_O_4_–β-cyclodextrin nanoparticles, and the effect of these prepared nanoparticles was evaluated in a chronic model of pentylenetetrazole-induced epilepsy. In comparison to free quercetin, quercetin-loaded magnetic nanoparticles (MNPs) reduced the behavioral signs of seizure, neuronal loss and astrocyte activation in a PTZ-induced kindling model more efficiently, which binds with the enhancement of water solubility and bioavailability of quercetin. Also, the solid lipid nanoparticle of quercetin, prepared by solvent evaporation method as predicted experimental designing patterns, was found to attenuate PTZ-induced neurocognitive impairments and ameliorate biochemical changes [199,200]. Drugs registered as first-line treatment in epilepsy, such as valproic acid, have multiple side effects, such as nephrotoxicity and prooxidative abilities. What makes quercetin a possible adjuvant to the therapy of epilepsy is its reno-protective and antioxidant action by reducing oxidative stress markers and quenching free radicals [142]. Another approach to combined management of epilepsy with inducing quercetin is to ameliorate depression associated with epilepsy dose-dependently. Elevation of indoleamine 2,3-dioxygenase (IDO) enzyme while using anticonvulsants, reliable for depressive symptoms, can be safely attenuated with oral administration of quercetin [201]. However, numerous studies indicate that quercetin has a narrow therapeutic window. Quercetin in higher doses significantly increases MDA levels in the hippocampus and cerebral cortex compared to the epileptic control group. That is because, at higher doses, quercetin can also promote pro-convulsant activity and prooxidative activity on the contrary mechanism [195]. In the penicillin-induced focal seizure rat model, lower quercetin doses had the highest anticonvulsant effect, significantly increasing the duration of latency (initial spike activity) and decreasing the spike frequency of the epileptiform activity compared to the control group [192]. Results were obtained by Kızılaslan et al.—low-dose quercetin reduced absence seizures by reducing proinflammatory cytokines and NO, but high-dose quercetin increased absence seizures by increasing the NO level [202]. In acute preclinical models of epilepsy by Nieoczym et al., quercetin was administered in different doses and at a dose range of 100–800 mg/kg IP was neither effective in the maximal electroshock test nor the PTZ IV seizure threshold model of epilepsy [203]. Studies investigating quercetin actions on epilepsy are summarized in Table 4.

## 5. Conclusions

Numerous dietary polyphenols exhibit actions beneficial for the central nervous system. Resveratrol, curcumin, luteolin and quercetin, are plant polyphenols naturally occurring in the human diet. Recent years of studies on animal models, cells, as well as clinical trials, confirm their beneficial activity. However numerous issues need to be solved. Actually, the strategies for efficient polyphenols administration and increasing bioavailability of polyphenols used in therapy should be developed. The use of nanoparticles to improve BBB overcoming, and the identification of mechanisms engaged in neural tissue distribution are the major goals for the future. Also, dietary recommendations concerning polyphenols-rich product intake in neurological disorders should be made, taking into account possible positive and negative effects.

## Figures and Tables

**Table 1:** Summarized results from studies investigating the use of resveratrol in the treatment of epilepsy.

Model	Intervention	Results	References
**Rats:** Sprague Dawley rats; Wistar rats; F334 rats weight range: 200–320 g seizures induction: PTZ 35 mg/kg/day; Lithium-pilocarpine 3 mEq/kg, i.p. and 30 mg/kg s.c.; kainate (KA) stereotactic injection; KA 2.5–5 mg/kg i.p. every hour; KA 10 µg	**Resveratrol:** dose range: 100 µM–60 mg/kg duration: 10 days; 35 days; 2 weeks form: raw; micronized; in liposomes; in glutathione-coated collagen nanoparticles way of administration: stereotactic injection bilaterally, 30 min after KA injection; intraperitoneal; orally; intragastrically; 1 h after SE episode every hour for 3 h; in acute TLE for 3 days, in silent and chronic TLE for 10 days after initial SE	**Seizures:** lower number of generalized seizures; lowered frequency of spontaneous seizures; micronized resveratrol decreased the occurrence of the tonic–clonic seizure stage seizures development slowed, raw resveratrol was not effective; resveratrol in liposomes is more effective anticonvulsant; encapsulated resveratrol was more effective	[144,145,146,147,148,149,150,152,153,154,156,157]
		**Neurons:** reduced neuron death in hippocampus; recovery of cognitive functions; prevention of glutamatergic neuron loss in hippocampus; improved neurogenesis;	
**Mice:** ICR mice; C57BL/6 mice seizure induction: intravenous NMDA (10 mg/mL) and 4-aminopyridine mg/mL); PTZ; Pilocarpine (340 mg/kg, s.c.) and methylscopolamine (1 mg/kg, s.c.)		**Inflammation:** reduced microglia activation	
**Fish:** Zebrafish (*Danio rerio*) seizure induction: 5 mM PTZ for 10 min		**Molecular changes:** reduced S100B in CSF and serum; increased expression of PGC-1α, NRF1, and Tfam; elevated NRF1 binding activity, COX1 level, and mtDNA amount; elevated mitochondrial biogenesis by activation of the PGC-1α signalling pathway; reduced caspase-3 activity; lower ROS production; resveratrol as a TLR3 blocker shows anti-epileptic effect	

**Table 2:** Summarized results from studies investigating the use of curcumin in the treatment of epilepsy.

Model	Intervention	Results	References
**Rats:** Sprague Dawley rats; Wistar rats weight range: 180–350 g seizure induction: KA (5 mg/kg); injection with PTZ (35 mg/kg) once daily; PTZ (40 mg/kg) for 30 days and on the 40th day; lithium (Li) and pilocarpine (Pc); PTZ (40 mg/kg) every second day for 30 days and on the 40th day; pilocarpine (380 mg/kg); KA (4 μg); FeCl_3_ injection; lithium chloride (125 mg/kg)/pilocarpine 20 (mg/kg)	**Curcumin:** dose range: 1 μg/mL–200 mg/kg duration: 24 days; 40 days; 3 days; 40 days; 5 weeks; 2 weeks; 14 and 28 days; 21 days form: raw; micronized; nanoparticles; encapsulated in chitosan-alginate-tripolyphosphate nanoparticles; in hydroxypropyl-b-cyclodextrin; in solid lipid nanoparticles (SLNs); in liposomes way of administration: curcumin pretreatment before KA stimulation; 3 days before Li and Pc; 30 min before PTZ	**Seizures:** suppression of spontaneous seizure-like events;	[158,159,160,161,162,163,164,165,166,167,168,169,170,171,172,173,174,175,176,177,178,179,180,181]
		**Neurons:** reduced neuronal loss in hippocampus; neuroprotection; inhibition of necroptosis; modulation of neurotransmitter level; SLNs increase neuroprotective activity of curcumin	
**Mice:** NMR mice; Swiss albino mice; C57BL/6 mice weight range: 20–35 g seizure induction: PTZ 36.5 mg/kg daily for 20 days; PTZ 35 mg/kg; KA (10 mg/kg)		**Inflammation:** lowered levels of pro-inflammatory cytokines and chemokines in hippocampus and cortex	
**Fish:** Zebrafish (*Danio rerio*) seizure induction: 3 mM PTZ		**Molecular changes:** suppression of the MAPK pathway; decreased levels of IL-6 and COX-2 in cultured astrocytes; reduced ROS level in neuronal cell line; reduction in IL-1 and NLRP3 levels; inhibited activation of NLPR3/inflammasome in epilepsy; downregulation of TNF-α; inhibited expression of GFAP and Iba-1; downregulation of CACNA1A and GABRD expression	
**Human:** 22 children (11 boys, 11 girls), 3–16 years old seizures status: at least 4 seizures during the last month			
**Cells:** human fetal astrocytes; neuronal cell line			

**Table 3:** Summarized results from studies on Luteolin’s effect on epilepsy.

Model	Intervention	Results	References
**Rats:** Wistar rats; Sprague-Dawley rats weight range: 150–250 g seizure induction: PTZ 80 mg/kg, in single dose i.p.; PTZ 35 mg/kg/day; 30 days, i.p., KA 15 mg/kg (i.p.)	**Luteolin:** dose range: 5–50 mg/kg duration: 2 weeks; 7–20 days form: raw; micronized way of administration: 30 min. before acute SE induction; together with PTZ; 30 min before KA injection	**Seizures:** reduced frequency; decreased severity and duration; prolonged latency between seizures	[182,183,184,185,186,187,188,189]
		**Neurons:** prevention of damage in hippocampus, protection from KA-induced neurotoxicity; increased hippocampal neurogenesis; increased dendritic spine maturation; restoration of cognitive functions	
**Mice:** Swiss albino mice; CDKL5 deficiency mice weight range: 18–25 g seizure induction: PTZ 100 mg/kg, (acute) or 35 mg/kg (chronic), i.p.		**Inflammation:** suppression of neuroinflammation	
**Fish:** Zebrafish (*Danio rerio*) seizures induction: PTZ-induced		**Molecular changes:** increased eNOS activity; stabilized iNOS and MMP2 activity; inhibition of the PTZ-activated TLR4/IκBα/NF-κB pathway and TNF-α, IL-6, IL-1β synthesis; increased Akt activation in the hippocampus	

**Table 4:** Summarized results from the studies on quercetin’s effect on epilepsy.

Model	Intervention	Results	References
**Rats:** Wistar rats; WAG/Rij rats weight range: 180–250 g seizure induction: intracortical penicillin injection (500 U/2.5 µL); PTZ 25 mg/kg i.p. for 28 days; PTZ 35 mg/kg/day for 15 days (i.p.)	**Quercetin:** dose range: 5–300 mg/kg duration: daily for 21 days; 5 days; 7 days form: solid lipid nanoparticles (SLN-Q); native; quercetin-loaded nano iron oxide particles coated with βCD and pluronic F68 polymer way of administration: 30 min before PTZ injections; at 7th day 30 min before KA injection	**Seizures:** decreased spike frequency of the epileptiform activity; decreased severity and frequency of seizures	[192,193,194,195,196,197,199,200,201,202,203]
		**Neurons:** attenuation of neurocognitive deficiency; quercetin in nanoparticles was more efficient in reduction in seizure behavior and neuron death	
**Mice:** BALB/c mice; Swiss albino mice weight range: 20–28 g seizure induction: KA 10 mg/kg (i.p.) in single dose; pilocarpine 360 mg/kg (i.p); PTZ (35 mg/kg, i.p.) in intervals 48 h; 6 Hz stimulation-induced psychomotor seizures; PTZ at day 5, 10, 15		**Inflammation:** decreased astrocyte activation; quercetin in low doses (25 mg/kg) reduced neuroinflammation (TNFα, IL-6, NO)	
**Fish:** Zebrafish (*Danio rerio*) seizure induction: PTZ exposition (7.5 mM for 3–4 min)		**Molecular changes:** scavenging of ROS; modulation of antioxidant enzymes activity; increased oxidative stress marker (MDA); prevented increase in β1 and β3 subunits of GABAA receptor; inhibited β1 and β3 subunits expression for 7 days after KA dose; in high doses quercetin increased NO	
